# Supplementary material for: Hog1-induced transcription of RTC3 and HSP12 is robust and occurs in cells lacking Msn2, Msn4, Hot1 and Sko1
Source: PLoS One. 2020 Aug 17;15(8):e0237540. doi: 10.1371/journal.pone.0237540 (PMC7430751; doi:10.1371/journal.pone.0237540)
Supplement: Attachment — Submitted filename: Peer review for PLOS ONE.pdf [file pone.0237540.s001.pdf]

The manuscript PONE-D-20-12974 entitled “Hog1-induced transcription of RTC3 and HSP12 is robust and occurs in cells lacking Msn2, Msn4, Hot1 and Sko1” by Engelberg et al. investigates the transcriptional regulation of four Hog1-target genes RTC3, HSP12, DAK1 and ALD3 in yeast.
What emerges from this work is that in all cases the factors Msn2,4 are required for expression but the cooperation of Hot1 and Sko1 is also important. RTC3 and HSP12, differently from DAK1 and ALD3, show a robust expression also in the absence of all the transcriptional regulators, suggesting the involvement of other factors as well as Hog1 itself. Authors observed that the yeast genetic background may influence transcriptional regulation of RTC3 and HSP12, but not of DAK1.
Proposed data surely required a big effort and represent a valuable contribution for individual promoter studies in different yeast strains genetic background, but the whole work presents several weaknesses, thus I suggest a major revision before publication in PLOS ONE.

Major points:

The importance of this study within the cellular context under physiological or stress conditions remains unfocused and should be better clarified. For example, some indications on the physiological function of the analyzed genes should be described and could also be discussed in the light of their different regulation.

The whole text for legends to Figures should be revised starting from the titles. The legend contents often overlap with results section and in general should be more concise.

Resolution for all the figures is very poor and should be improved.

Also legends to the graphs are too long and should be simplified (+Met; -Met; 0.7 M NaCl; control) with more detailed explanation in the text legends.

Statistical analysis should be added where required.

The manuscript structure is confusing: division in subparagraphs is unclear, figure legends and results are mixed up.

The cited literature seems incomplete: for example, the proposed role of RTC3 in response to high sugar stress and its relations to HSPs proteins is not mentioned (PMID:21247650).

Minor points:

The order of authors is unclear: there is no correspondence between the first page of the submission and the first page of the manuscript. Who is the first author? Who is the corresponding author?

Line 74, change “activates” with “activate”

Lines 189-191 Reformulate these two sentences

Line 291 Correct “background”
