# Supplementary material for: Hog1-induced transcription of RTC3 and HSP12 is robust and occurs in cells lacking Msn2, Msn4, Hot1 and Sko1
Source: PLoS One. 2020 Aug 17;15(8):e0237540. doi: 10.1371/journal.pone.0237540 (PMC7430751; doi:10.1371/journal.pone.0237540)
Supplement: Attachment — Submitted filename: Response to Reviewers.docx [file pone.0237540.s002.docx]

Dr. Juan Mata
Academic Editor, PLOS ONE

Re: PONE-D-20-12974

Dear Dr. Mata,

Many thanks for your e-mail of May 21 that included the reviewers’ comments to our manuscript. We have prepared a revised version in which we addressed all comments and hope that this version will merit publication in PLoS One. A point-by-point list of answers to reviewers’ comments is appended below. Original uncropped gels were uploaded, as requested, as a file entitled ‘uncropped gels’.

Sincerely,

David Engelberg

A point-by-point list of response to reviewers’ comments

Reviewer 1:

1. The reviewer asks to show the data regarding STRE-LacZ activity – we thus added this data as an upper panel in Figure 1F.

2. The reviewer comments about sequences, which were underlined in Fig. 1E, and were not referred to properly in the text – we agree with the reviewer that the text in these lines and its relatedness to the figure were confusing. We now removed all lines from the sequence in Figure 1E and simplified the explanation in lines 330-339 (in the new version).

3. The reviewer asks whether qRT-PCR analysis was performed in three repeats just as β-galactosidase assays were – in the new version we more explicitly state this matter (lines 194-195) and explain the related statistical analysis (lines 195-197).

4. The reviewer noted that the primers Table is missing. This must have been a technical problem. In any case, primers appeared in the Table were now simply added them as a lists in Materials and Methods (lines 146-160; 188-192).

5. The verb was added: cloned. Now in line 213.

6. The letter D was changed to E. We thank the reviewer for his/her so rigorous reading.

7. 1D was changed to 1E. Thanks again.

8. The word backupping was changed to backup factors (now line 348).

Reviewer 2:

1. The reviewer suggests to add more information on the physiological functions of the genes studied – although the study deal with transcription of these genes, we accepted the recommendation and added: 1) information on the enzymatic activity of each of the gene products (in lines 212-219). Some information on the physiological functions of each gene products is provided now in lines 320-322, 418-420, 468-469, 510-512.

2. Figure legend and titles of legends were shortened.

3. Figures resolutions were improved.

4. Legends of graphs were shortened.

5. Many more references were added, including the one recommended by the reviewers.

Added references are: 31, 33, 34, 38, 40, 41.

6. The order of authors is determined by the website – corrected now.

7. The letter s was added.

8. The word background is now spelled correctly.
