# Supplementary material for: Hog1-induced transcription of RTC3 and HSP12 is robust and occurs in cells lacking Msn2, Msn4, Hot1 and Sko1
Source: PLoS One. 2020 Aug 17;15(8):e0237540. doi: 10.1371/journal.pone.0237540 (PMC7430751; doi:10.1371/journal.pone.0237540)
Supplement: Attachment — Submitted filename: Response to Reviewers2.docx [file pone.0237540.s003.docx]

Dr. Juan Mata
Academic Editor, PLOS ONE

Re: PONE-D-20-12974

Dear Dr. Mata,

Many thanks for your e-mail of July 23^rd^ that included the reviewers’ comments to our revised manuscript. We have prepared a revised version in which we addressed all comments and hope that this version will merit publication in PLoS One. A point-by-point list of answers to reviewers’ comments is appended below.

Sincerely,

David Engelberg

A point-by-point list of response to reviewers’ comments

Reviewer 2:

**1.** The reviewer raises the issue of figures resolution. I fully agree with the reviewer’s comment that was also raised in the first reviewing round. We must note that we upload high quality figures that passed all tests of the *PLoS One* submission system. The reduction in quality occurs when the PDF file in created. I noticed this problem even before the reviewer’s comment and approached *PLoS One* assistance system. Here is the correspondence that clearly suggest that the issue is in the hands of PLoS One editorial staff:

Dear Dr. Engelberg,
Thank you for your email.

I'm sorry to hear that you're upset about the quality of your figures in the submission PDF. However, please be assured that the submission PDF is for reviewing purposes only and does not reflect the appearance of the final publication, should your manuscript be accepted. Instead, the figures will be typeset at the resolution of your original submitted figures.
Please also note that editors and reviewers will have access to the original source files you’ve provided via the blue download link in the top right corner of the PDF rendering of your figure.

For more information about our figure requirements, please see (https://journals.plos.org/plosone/s/figures).
Thanks for considering PLOS ONE for your work. Please feel free to contact us if you have other questions.

Best,
Eoin O'Connor

Editorial Office
PLOS | plos.org
Empowering researchers to transform science
1160 Battery Street, Suite 225, San Francisco, CA 94111

Case Number: 06690049
ref:_00DU0Ifis._5004P1Bd6nK:ref

--------------- Original Message ---------------
**From:** David Engelberg [engelber@mail.huji.ac.il]
**Sent:** 29/06/2020 15:42
**To:** plosone@plos.org
**Subject:** Re: PLOS ONE Decision: Revision required [PONE-D-20-12974] - [EMID:501e99ac72418e0f]

Hello,
I have just uploaded the revised manuscript at the website.
Two matters bother me:
A. The list of authors as appears in the submission manager (with me as the first author) is not the correct one - the correct one is as appears in the manuscript title page with Bai Chen as the first author [one of the reviewers commented on it]. I hope that if the paper is accepted the proper order of authors will appear on it.
B. Although I ran all figures through the Apex.PACE system, as requested, and although the quality is quite high as appears in the files, figures look less good on the final PDF file. I hope that, if the paper is accepted, this will not be the quality of the final publication.
Thanks for dealing with our manuscript.
Sincerely,

David Engelberg

**2.** The reviewer suggest to further shorten the titles of figure legends and to add description of growth conditions and RNA extraction. We accepted the comments. All figure legend titles have been shortened: lines 337, 438, 490 and 535. Growth conditions are now added (lines 141-151) and the kit used for RNA extraction is mentioned now in line 147.
